# International Society of Laser Proctology Position Paper on SiLaC and EPSiT/SiLaC in the Management of Pilonidal Sinus Disease

**DOI:** 10.1002/lsm.70071

**Published:** 2025-10-13

**Authors:** Mustapha Ouali, Gonzalo P. Martin‐Martin, Salih Avdicausevic, Amine Alam, Pavel Istok, Ints Brunenieks, Vladimir Dobricanin, Vincent de Parades, Jiong Wu, Peter C. Ambe

**Affiliations:** ^1^ Proctolaser Clinic Sfax Tunisia; ^2^ Hospital Recoletas Salud López Cano Cádiz Spain; ^3^ COC‐Ingolstadt‐Süd (Chirurgisch Orthopädisches Centrum) Ingolstadt Germany; ^4^ Service de Proctologie Médico‐Chirurgicale, Hôpital Paris Saint‐Joseph Paris France; ^5^ Proktovena by Dr. Ištok Bratislava Slovakia; ^6^ Department of Proctology and Phlebolog Clinic Aizkraukle Latvia; ^7^ Clinic Center of Montenegro (KCCG), Faculty of Medicine University of Montenegro Podgoric Montenegro; ^8^ Department of Coloproctology, Yueyang Hospital of Intergrated Traditional Chinese and Western Medicine Shanghai University of TCM Shanghai China; ^9^ Department of Surgery Klinik Oberwart Oberwart Austria; ^10^ Faculty of Health Witten/Herdecke University Witten Germany

**Keywords:** EPSiT, laser, pilonidal cyst, pilonidal sinus, pilonidal sinus disease, SiLaC

## Abstract

**Objectives:**

Minimally invasive treatment of pilonidal sinus disease (PSD) via sinus tract laser closure (SiLaC) and endoscopic pilonidal sinus treatment (EPSiT) is being increasingly employed to manage PSD. Despite its wide adoption amongst colorectal surgeons in many nations, there still exists a wide heterogeneity in surgical technique. The International Society of Laser Proctology (ISoLP) was created by experts in laser proctology has the goal of improving laser‐based interventions in coloproctology with the aim of improving patient care in this specialized area.

**Methods:**

This position paper is based on the limited evidence from available literature and largely on the expert opinion of ISoLP members with extended expertise in SiLaC and EPSiT.

**Results:**

ISoLP experts in SiLaC and EPSiT procedures have suggested 10 statements to aid in homogenizing the SiLaC procedure alone and in combination with EPSiT, with the goal of improving treatment outcomes.

**Conclusion:**

The 10 ISoLP position statements have a potential of shaping and homogenizing laser‐assisted management of PSD.

## 1 Introduction

Pilonidal sinus disease (PSD) is a chronic inflammatory condition of the subcutaneous tissue, most often in the midline of the natal cleft, characterized by one or more epithelialised sinus tracts that typically contain hair and keratinous debris [1, 2]. Once considered congenital, PSD is now viewed as an acquired condition in which invading hairs, driven into the skin by friction, pressure and suction during sitting or movement, trigger a foreign‐body granulomatous response that can manifest as an acute abscess or evolve into a chronic suppurative process with granulation tissue, intermittent discharge, and recurrent infection [3].

PSD predominantly affects young adults, peaking between 15 and 30 years of age with a male‐to‐female ratio of roughly 3–4:1 [4]. Risk factors include coarse body hair, sedentary lifestyle, prolonged sitting, a deep natal cleft, obesity, local trauma, poor hygiene, and positive family history. Incidence estimates range from 26 to 70 cases per 100,000 population annually [4]. Recurrence, especially when risk factors persist or treatment is inadequate, adds substantially to the burden of disease through impaired quality of life and increased healthcare utilization [2].

Over the last decade, laser‐based surgery has increasingly been adopted worldwide as a preferred technique for PSD [5, 6]. Sinus laser‐assisted closure (SiLaC) uses laser energy to treat PSD [7]. SiLaC can be combined with endoscopic pilonidal sinus treatment (EPSiT), another minimally invasive technique, as complementary procedures for the management of PSD. The current literature testifies a high rate of success for both techniques with acceptable morbidity rates [8, 9]. However, current evidence is limited to small retrospective series. This limitation applies to nearly all laser‐based procedures in proctology [6, 10].

In October 2024, the International Society of Laser Proctology (ISoLP) was created by experts in laser‐assisted surgery in proctology with the goal of improving the technique as a central aspect of patient care (www.isolp.org). This paper represents ISoLP's effort in shedding some light on these two minimally invasive interventions for PSD. This position paper is based on evidence from available literature and largely on the expert opinion of ISoLP members with extensive experience with laser management of PSD. A total of 10 position statements (P1–P10) have been formulated by a dedicated ISoLP expert group for the management of PSD via SiLaC/EPSiT.

## 2 Diagnosis of PSD

The diagnosis of PSD is primarily clinical, based on focused physical examination. Typical findings include one or more midline or paramedian pits in the natal cleft, sometimes with purulent discharge, erythema, or local swelling. Acute presentations may show a fluctuant abscess, whereas chronic cases often exhibit intermittent discharge and induration. Important differentials include perianal fistula, hidradenitis suppurativa, and dermoid cyst.

PSD can be classified according to presentation, extent, and complexity. Most grading systems distinguish acute from chronic disease, the latter subdivided into simple and complex forms. Simple disease comprises a single midline pit with a limited tract, whereas complex disease includes multiple openings (often lateral to the midline), extensive subcutaneous tracts or recurrent episodes. Frameworks such as the German National Guideline incorporate the number of sinuses, lateral extension, and prior surgery to stratify severity and guide management, yet no universally accepted classification exists, hampering comparison of study outcomes and treatment protocols [11].

P1—Diagnosis of PSD is primarily clinical following focused physical examination. Typical findings include a pit in or around the midline of the natal cleft. The acute situation is characterized by a collection/abscess, while the chronic scenario may present with intermittent secretions.

## 3 Imaging Before SiLaC/EPSiT

In most primary, uncomplicated pilonidal sinus cases, imaging is not usually required prior to surgery; careful clinical inspection usually provides sufficient information for planning SiLaC or EPSiT [12].

**Figure 1:**
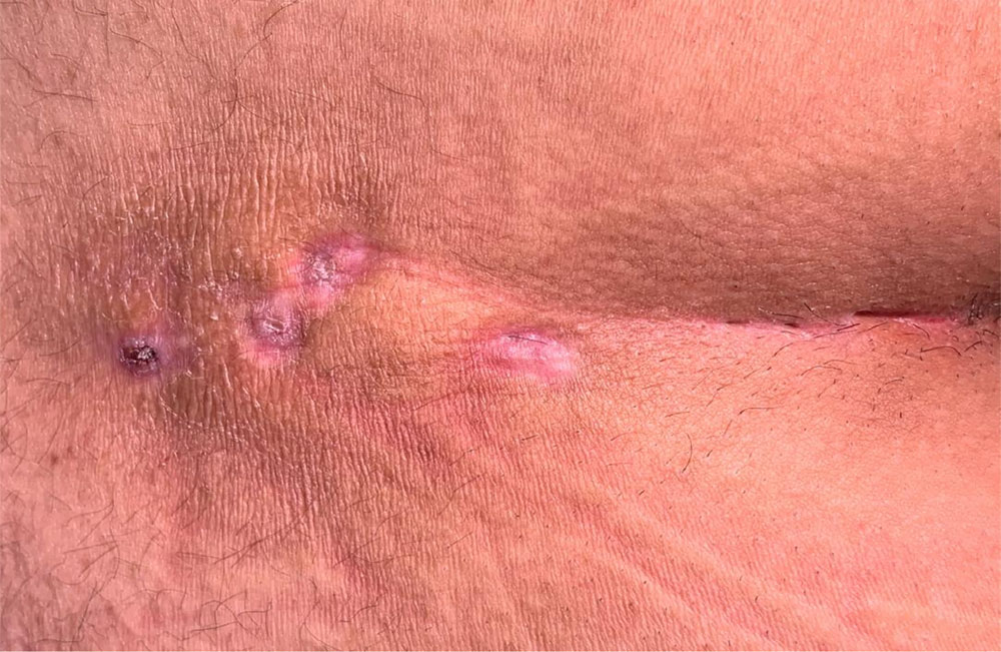
Preoperative view of chronic pilonidal sinus with multiple cutaneous pits. Courtesy of Prof. Jiong Wu.

Imaging becomes advisable when the disease is complex with multiple pits, recurrent or long‐standing, when pits are atypically located or laterally displaced, or when an abscess or deep extension is suspected [12, 13]. Figure 1 shows a preoperative clinical image of a complex PSD with multiple midline and lateral pits. Imaging prior to surgery in such cases should complement physical examination and should be considered in the decision‐making with regard to treatment options and expected outcomes.

**Figure 2:**
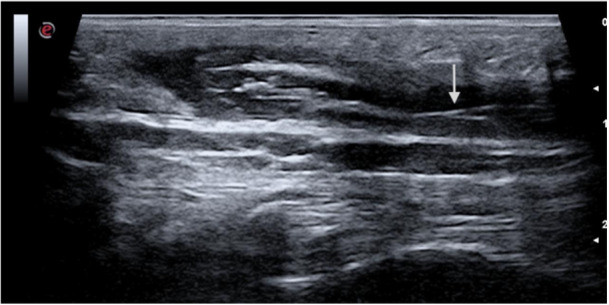
High frequency ultrasound of the natal cleft showing a collection and hair follicle in the sinus (arrow).

High‐frequency ultrasound allows for bedside mapping of superficial channels and detection of occult collections with a reported sensitivity of 80%–90% [14, 15]. Figure 2 demonstrates a preoperative ultrasound scan with clear identification of hair (arrow) in the sinus.

**Figure 3:**
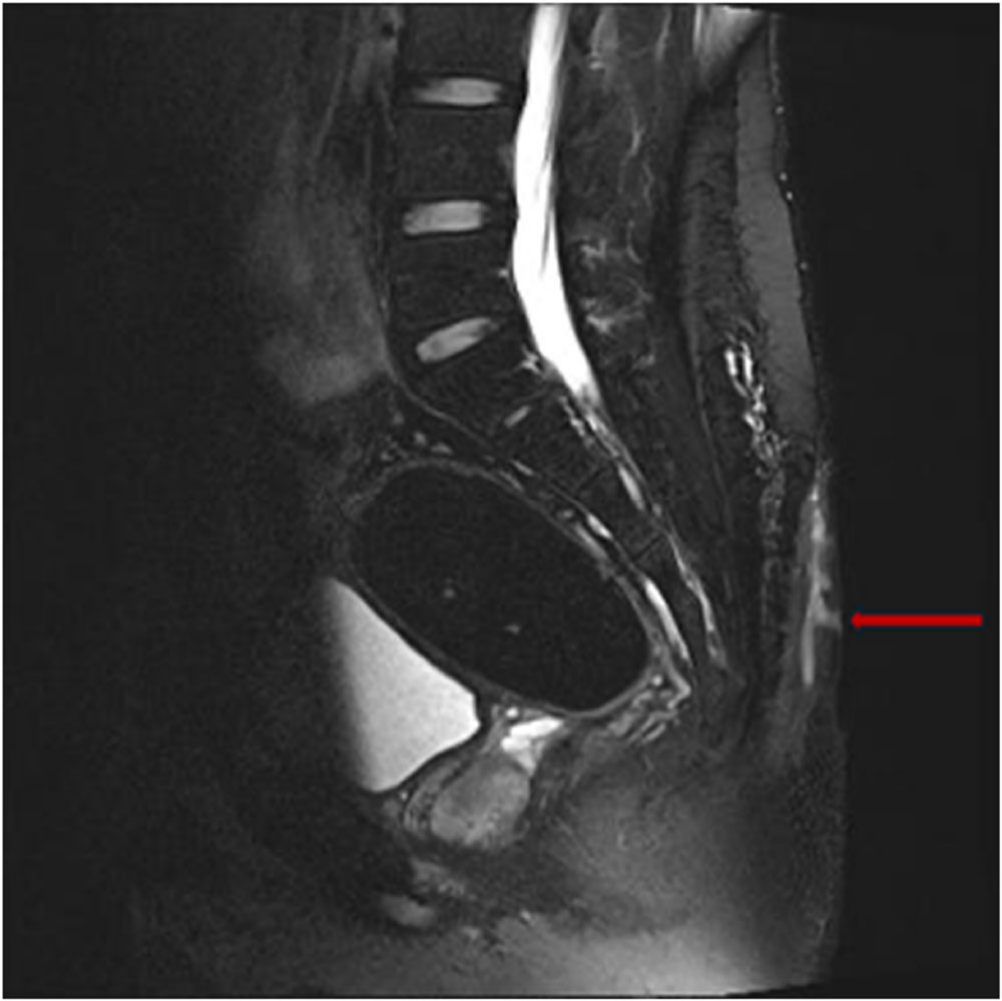
Sagittal MRI image demonstrating a complex PSD (red arrow). Courtesy of Prof. Jiong Wu.

Magnetic‐resonance imaging offers superior soft‐tissue resolution and remains the cross‐sectional modality of choice for extensive or multiloculated networks, albeit a higher cost and with limited availability [16]. Computed tomography confers ionizing radiation and is reserved for patients in whom MRI is contraindicated or unavailable [16, 17]. Figure 3 shows the corresponding MRI image to the case of complex PSD demonstrated in Figure 1.

When an endoscopic approach (EPSiT) is planned, the fistuloscope provides real‐time visualization that often obviates routine imaging. Nevertheless, ISoLP suggests considering preoperative imaging via ultrasound or MRI in complex, recurrent, or multicavity disease to anticipate tract length and branching [13, 16]. ISoLP, therefore, does not encourage routine imaging before SiLaC/EPSiT but rather supports a selective use of preoperative imaging based on clinical complexity.

P2—SiLaC and EPSiT can be routinely performed without prior imaging. Imaging may be necessary in recurrent disease and may be considered in cases with atypical pit location. Ultrasound or MRI are appropriate imaging modalities in PSD.

### 3.1 Laser‐Assisted Pilonidal Sinus Therapy (SiLaC ± EPSiT)

#### 3.1.1 Management, Outcomes, and Complications

Conventional surgical treatment techniques, such as wide excision with secondary healing [18], excision with primary midline or off‐midline closure [19], an array of flap reconstructions (e.g., Limberg, Karydakis, Bascom cleft‐lift) [20], and various modifications have long been employed to manage PSD. Also, nonsurgical options like chemical ablation with phenol injection [21] and pit picking [22] are also practiced worldwide. Excision and flap reconstruction procedures carry substantial morbidity, necessitate protracted wound‐healing periods, and remain burdened by notable recurrence rates [23].

The advent of minimally invasive modalities, most notably sinus laser closure (SiLaC) [23] and endoscopic pilonidal sinus treatment (EPSiT) [24], and pit picking [22], alongside phenol injection [21], has reshaped the management of PSD by introducing minimally invasive tissue‐sparing options [5]. These treatment options can be complemented by a hybrid technique combining EPSiT and SiLaC (E‐SiLaC). E‐SiLaC uses the advantages of EPSiT to overcome the lack of direct visualization in SiLaC, as its most serious limitation.

P3—SiLaC, EPSiT, and E‐SiLaC belong to minimally invasive, tissue‐preserving management techniques for PSD.

### 3.2 Indications and Contraindications for SiLaC and E‐SiLaC

For both SiLaC and E‐SiLaC, an acute abscess represents an absolute contraindication  [20]. Coagulopathy, severe immunosuppression, and inability to comply with postoperative care are relative contraindications  [25].

*Absolute contraindication*

- Acute pilonidal abscess [26].

*Relative contraindications*

- Multiloculated or highly recurrent disease [13, 27].
- Coagulopathy [25].
- Severe immunosuppression [25].
- Inability to comply with postoperative care, including hair‐removal regimen [25].

P4—ISoLP does not encourage SiLaC/E‐SiLaC in the acute setting; acute abscess/collections with cellulitis constitute absolute contraindications.

### 3.3 SiLaC Procedure

In the SiLaC procedure, a 1470 nm 360° radial‐emitting laser fiber is advanced into each sinus tract and slowly withdrawn at ≈1 mm/s while delivering continuous laser energy [24, 28]. The laser power should be individually chosen depending on the individual case, for example, low power like 8 watts for skinny patients and up to 10–12 watts for thick‐skinned and obese patients. The laser energy induces photothermal ablation of the epithelial lining, protein denaturation, immediate cavity shrinkage, and tract obliteration, followed by secondary inflammatory remodeling [24, 28].

SiLaC is feasible under local, regional, or general anesthesia, with the patient in the prone jackknife or lateral position [24]. Skin preparation comprises shaving, thorough disinfection of the natal cleft and lower back, followed by sterile draping. Pit access is usually after enlargement with a 3–8 mm biopsy punch [29]. Alternatives include blunt exploration with a mosquito clamp [30] or a cross‐incision [31]. Trapped hairs and granulation tissue are removed as in pit picking; curettage, debridement and saline irrigation are optional [32].

The radial fiber is inserted into the sinus/tract and withdrawn at a constant speed of about 1 mm/s while firing the laser along every branch [28]. ISoLP suggests gentle external compression with wet, cooled gauze during energy delivery to enhance fiber‐wall contact and dissipate heat; intumescent anesthesia around the tract may further augment this effect [24].

P5—Standard SiLaC comprises of pit‐picking (with or without debridement) plus laser ablation/obliteration using a 360° fiber, under any form of anesthesia. ISoLP strongly advises protecting the overlying healthy skin (e.g., cooled gauze) and applying gentle compression to improve tract obliteration.

### 3.4 Endoscopic Pilonidal Sinus Treatment (EPSiT) in Combination With SiLaC (E‐SiLaC)

EPSiT uses a slim fistuloscope (≈3.3 mm × 4.7 mm), which is introduced into the sinus, permitting direct visualization, mapping, irrigation, and cleansing [33]. Through its working channel, a brush, grasper, or radial laser fiber can be introduced to debride and ablate the sinus/tract. Introducing the radial fiber enables endoscopic SiLaC (E‐SiLaC), thus providing real‐time visual control and mitigating SiLaC's blind‐spot limitation [33, 34].

ISoLP particularly encourages E‐SiLaC for recurrent or complex sinus networks and for cases of long‐standing discharge to explore persistent tracts [33].

P6—ISoLP particularly encourages E‐SiLaC in recurrent or complex sinus networks.

### 3.5 Outcomes

Table 1 presents the healing rates for SiLaC and E‐SiLaC. The percentages reflect pooled estimates from recent systematic reviews and multicenter cohorts. Both SiLaC and E‐SiLaC demonstrate high healing rates, with E‐SiLaC showing slightly higher values in some series.

P7—SiLaC and E‐SiLaC have comparable success rates. ISoLP therefore suggests E‐SiLaC for selected cases, as in P6.

**Table 1:** Summary of healing and failure rates for SiLaC and E‐SiLaC.

Outcome	SiLaC	E‐SiLaC
Primary healing rate	75%–94% [12, 35, 36]	85%–96% [13, 37]
Failure/recurrence rate	8%–25% [12]	4%–15% [13]

### 3.6 Complications and Morbidity

Overall, morbidity after both procedures is quite low (pooled 6%–12%) and mainly minor (Clavien–Dindo I–II). Commonly reported complications are presented in Table 2. No Clavien–Dindo IV or V events have been reported [38, 39].

P8—SiLaC and E‐SiLaC are both associated with a low risk of minor complications; patients can therefore be counseled similarly for either procedure.

**Table 2:** Summary of complications following SiLaC/E‐SiLaC.

Complication	Clavien–Dindo grade	SiLaC (%)	E‐SiLaC (%)
Transient postoperative pain requiring oral analgesics	I	30%–45% [38]	25%–40% [39]
Localized wound infection requiring oral antibiotics	II	3%–8% [38]	2%–6% [39]
Seroma/hematoma requiring needle aspiration	IIIa	1%–3% [38]	0%–2% [39]
Abscess or persisting sinus requiring surgical drainage/redo surgery (SiLaC/E‐SiLaC)	IIIb	4%–10% [38]	2%–6% [39]

### 3.7 Hair Removal and Recurrence

Loose or broken hairs entering the natal cleft are a key cause of primary PSD and recurrence. In a recently published systematic review by Basso et al. systematic postoperative depilation, preferably using a laser, substantially reduced the risk of recurrence by about 60% [3]. In another publication by J. L. Bascom and B. R. Bascom, the 3‐year recurrence rate dropped from 18%–23% to 5%–8% in a cohort following diode‐laser hair removal [4]. The results of a recently published randomized controlled trial by Minneci and colleagues demonstrated a statistically significant reduction in recurrence of PSD following postoperative laser epilation in adolescents and young adults in comparison to standard of care (10.5% vs. 33.6%) 12 months after surgery [40]. This trend could be confirmed in a systematic review or RCTs from 2024 by Muscat et al. [8]. Although shaving and creams can be employed as means of hair removal, both are inferior to laser epilation. In a systematic review by Pronk and colleagues, recurrent was lowest following laser epilation (9.3%) in comparison with razor shaving or cream depilation (19.7%) [41].

A meta‐analysis of 7 cohort studies with 1248 patients showed a close to 35% relative recurrence reduction with regular hair removal [37]. Laser epilation provides the most durable clearance and fewer folliculitis episodes compared with shaving or waxing [40].

ISoLP suggests initiation of hair removal after wound healing is complete (~2–4 weeks post‐SiLaC/EPSiT), repeating this every 4–6 weeks for ~6–8 sessions.

P9—ISoLP suggests long‐term hair removal, preferably via laser epilation, to reduce recurrence risk after PSD treatment. Timing of hair removal should be after complete wound healing following SiLaC/E‐SiLaC (2–4 weeks).

### 3.8 Cost of SiLaC Versus E‐SiLaC

PSD commonly affects young adults; thus, the choice of treatment has some economic impact. Extensive surgery involves a long operating time and prolonged work absence. Minimally invasive surgery offers shorter procedural time and quicker return to work. EPSiT/E‐SiLaC incurs higher material cost and longer operating time, yet shows success rates comparable to SiLaC. Consequently, ISoLP advises selective use of E‐SiLaC [29].

P10—Because of higher procedural cost, ISoLP recommends limiting E‐SiLaC to well‐selected cases as defined in P7.

## 4 Conclusion

These 10 ISoLP position statements have a potential of shaping and homogenizing laser‐assisted management of PSD.

## Ethics Statement

This manuscript was written in accordance with ethical standards. This is a “Not Human Subjects Manuscript”. As such, IRB approval was not required.

## Consent

This is a “Not Human Subjects Manuscript”. As such, informed consent was not required.

## Conflicts of Interest

The authors declare no conflicts of Interest.
